# Automated identification of atrial fibrillation from single-lead ECGs using multi-branching ResNet

**DOI:** 10.3389/fphys.2024.1362185

**Published:** 2024-04-09

**Authors:** Jianxin Xie, Stavros Stavrakis, Bing Yao

**Affiliations:** ^1^ School of Data Science, University of Virginia, Charlottesville, VA, United States; ^2^ Health Sciences Center, University of Oklahoma, Oklahoma City, OK, United States; ^3^ Department of Industrial and Systems Engineering, University of Tennessee at Knoxville, Knoxville, TN, United States

**Keywords:** convolutional neural network, residual network, wavelet transform, multi-branching outputs, ECG signal analysis, imbalanced data, atrial fibrillation

## Abstract

**Introduction:** Atrial fibrillation (AF) is the most common cardiac arrhythmia, which is clinically identified with irregular and rapid heartbeat rhythm. AF puts a patient at risk of forming blood clots, which can eventually lead to heart failure, stroke, or even sudden death. Electrocardiography (ECG), which involves acquiring bioelectrical signals from the body surface to reflect heart activity, is a standard procedure for detecting AF. However, the occurrence of AF is often intermittent, costing a significant amount of time and effort from medical doctors to identify AF episodes. Moreover, human error is inevitable, as even experienced medical professionals can overlook or misinterpret subtle signs of AF. As such, it is of critical importance to develop an advanced analytical model that can automatically interpret ECG signals and provide decision support for AF diagnostics.

**Methods:** In this paper, we propose an innovative deep-learning method for automated AF identification using single-lead ECGs. We first extract time-frequency features from ECG signals using continuous wavelet transform (CWT). Second, the convolutional neural networks enhanced with residual learning (ReNet) are employed as the functional approximator to interpret the time-frequency features extracted by CWT. Third, we propose to incorporate a multi-branching structure into the ResNet to address the issue of class imbalance, where normal ECGs significantly outnumber instances of AF in ECG datasets.

**Results and Discussion:** We evaluate the proposed Multi-branching Resnet with CWT (CWT-MB-Resnet) with two ECG datasets, i.e., PhysioNet/CinC challenge 2017 and ECGs obtained from the University of Oklahoma Health Sciences Center (OUHSC). The proposed CWT-MB-Resnet demonstrates robust prediction performance, achieving an F1 score of 0.8865 for the PhysioNet dataset and 0.7369 for the OUHSC dataset. The experimental results signify the model’s superior capability in balancing precision and recall, which is a desired attribute for ensuring reliable medical diagnoses.

## 1 Introduction

Cardiovascular diseases have been the leading cause of mortality globally. The World Health Organization (WHO) states that about 17.9 million people perish due to cardiovascular disease each year (World Health Organization, 2024), contributing 32% to the worldwide death toll (University of Washington, 2024). Atrial fibrillation (AF) is the most common cardiac arrhythmia caused by uncoordinated electrical activities in the atria (Nesheiwat et al., 2023). Although AF itself does not lead to a lethal condition, it will substantially increase the risk of catastrophic diseases such as heart failure, stroke, and sudden death (Lubitz et al., 2013; Bernstein et al., 2021). The prevalence of AF plagues over 2.7 million people in the United States, and this number is estimated to rise to 12.1 million in 2030, as the population ages (Colilla et al., 2013). In healthcare practice, the electrocardiogram (ECG) is a cost-effective and noninvasive medical approach to record the electrical signals on the body surface as a reflection of cardiac health conditions (Yao and Yang, 2016; Yao and Yang, 2020; Yao et al., 2021; Xie and Yao, 2023).

Historically, the utilization of ECG for cardiac monitoring has been substantially constrained by the need for expensive equipment and the involvement of specialized medical doctors to interpret complex ECG recordings. However, recent advancements in portable ECG sensors, such as the AliveCor (aliveCor, 2024), AD8232 (Analog Devices, 2024), and consumer-grade devices like the smartwatch (Isakadze and Martin, 2020), have revolutionized the way to detect heart abnormalities. These portable devices now enable the capture of high-fidelity ECG signals outside of traditional clinical settings. While multi-lead ECGs provide comprehensive cardiac activity information, single-lead ECGs make cardiac monitoring more accessible and less obtrusive for long-term rhythm surveillance or frequent measurements (Abdou and Krishnan, 2022). This is especially valuable in ambulatory settings, home monitoring, and situations where rapid and non-invasive monitoring is desired. Single-lead ECGs offer a simplified yet effective method for the early detection of AF and other cardiac anomalies (Boriani et al., 2021).

In conjunction with advanced sensing technologies, there has been a parallel development in machine learning methodologies. Given the prevalence of AF, a significant number of machine learning models have been developed specifically for the task of distinguishing AF from normal heart rhythms. Traditional machine learning models focus on extracting morphological features and heart rate variability from ECG signals to detect AF, which depends heavily on manual feature engineering (Ye et al., 2012; Da Silva-Filarder and Marzbanrad, 2017; Athif et al., 2018). Deep Neural Network (DNN), which does not require explicit feature engineering, is another powerful tool that has achieved promising results in data-driven disease detection. Various DNN-based models such as convolutional and recurrent neural networks (i.e., CNNs, RNNs) have been designed for AF detection and outperformed conventional machine learning methods (Andreotti et al., 2017; Schwab et al., 2017; Gao et al., 2021). Despite the performance improvement achieved by DNNs in detecting AF with single-lead ECG, there remains potential for further prediction enhancements. Four major challenges remain to be tackled: 1) ECG recordings collected from clinics are often in Protable Document Format (PDF). An effective preprocessing procedure is needed to retrieve digital ECG signals from the PDFs before being fed to the machine learning models. 2) ECG signals are generally composed of a wide spectrum of frequency components. DNN models built upon raw ECG time series may not fully exploit the time-frequency information inherent in the signals. 3) Note that the learning capacity for a DNN often increases when the network goes deeper. However, the deeper structure can result in gradient dissipation problems, leading to unsatisfactory prediction performance. 4) Data-driven AF detection also suffers from the common issue of imbalanced data in machine learning (e.g., AF samples are much less compared to normal ECGs). The classifier directly built from the imbalanced data will generate biased and inaccurate predictions.

In this paper, we develop an automatic AF detector based on continuous wavelet transform (CWT) and 18-layer Residual Neural Network (ResNet18) with a multi-branching structure (CWT-MB-ResNet). We first develop a preprocessing procedure to extract ECG signals from ECG PDFs and leverage the CWT to transform the extracted signals into the time-frequency domain. Second, ResNet18 is engaged to alleviate the gradient dissipation problem in deep-structured networks, allowing it to learn deeper features from 2D time-frequency images and achieve better performance. Finally, we propose to incorporate a multi-branching output structure adapted from our prior work (Wang and Yao, 2021) into the ResNet to deal with the issue induced by the imbalanced dataset in AF identification. The multi-branching technique exempts artificial data augmentation and does not require any preassumptions in solving the imbalanced data issue. The performance of the proposed framework is evaluated by two real-world datasets: PhysioNet/CinC challenge 2017 (Goldberger et al., 2000; Clifford et al., 2017) and ECG data obtained from the University of Oklahoma Health Sciences Center (OUHSC). Experimental results show that our CWT-MB-ResNet significantly outperforms existing methods commonly used in current practice.

The rest of this paper is organized as follows: Section 2 presents the literature review of existing data-driven methods for AF detection. Section 3 introduces the data processing details and the proposed prediction method. Section 4 shows the experimental results in AF identification. Section 6 concludes the present investigation.

## 2 Research background

Traditional machine learning approaches focus on the extraction of ECG morphological features (De Chazal et al., 2004) and heart rate variability information (Park et al., 2009) to identify AF conditions. Those methods are mostly in light of two aspects of AF-altered ECG characteristics: 1) the absence of distinct P waves, which are replaced by irregular fibrillatory waves or F waves as oscillations in low amplitude around the baseline (Ladavich and Ghoraani, 2015); 2) irregular R-R intervals (Oster and Clifford, 2015). Multiple feature-based automation techniques have been proposed to classify AF-altered ECGs, such as linear discriminant analysis (De Chazal et al., 2004), support vector machine (Billeci et al., 2017; Islam et al., 2017), independent component analysis (Ye et al., 2012). When there exists a high level of noise or faulty detection, the performance of feature-extraction methods that solely study the P wave deteriorates significantly due to the chaotic signal baseline introduced by the noise (Larburu et al., 2011). Most R-R interval-based methods (Tateno and Glass, 2001; Lian et al., 2011) usually require long ECG segments to detect AF episodes, and become ineffective when it comes to short ECG signals (less than 60s) or in the presence of significant sinus arrhythmia or frequent premature atrial contractions (Xia et al., 2018). Moreover, traditional methods require a separate feature extraction process before feeding the data into the classifier, as well as manually establishing the detection rules and threshold. This can be computationally expensive and may not generalize well when applied to a larger population.

In the past few decades, deep learning or deep neural network (DNN) has emerged as a powerful tool for pattern recognition that can learn the abstracted features from complex data and yield state-of-the-art predictions (Mousavi et al., 2019; Xie and Yao, 2022a; Xie and Yao, 2022b; Chen et al., 2022; Wang et al., 2022). As opposed to traditional machine learning, deep learning presents strong robustness and fault tolerance to uncertain factors, which makes it suitable for beat and rhythm classification from ECGs (Tutuko et al., 2021). Moreover, existing research has indicated that deep learning methods demonstrate more efficient and more potent predictive power than classical machine learning methods for AF identification (Cai et al., 2020; Murat et al., 2021). There has been a significant surge in leveraging deep learning for AF detection using single-lead ECGs, showing promising potential in enhancing diagnostic accuracy. We summarized four commonly used network structures in discerning AF samples using single-lead ECGs:

1) **Convolutional neural networks (CNNs):** CNNs, specifically 1-dimensional CNNs (1D-CNNs), have been widely applied to extracting hierarchical features from ECG data for distinguishing AF from normal heart rhythms (Andreotti et al., 2017; Fan et al., 2018; Lai et al., 2019; Phukan et al., 2023). For example, Andreotti et al. Andreotti et al. (2017) balanced the PhysioNet/CinC 2017 dataset by augmenting AF samples from various sources to address the class imbalance issue. They employed a ResNet model with 34 convolutional layers for AF detection, achieving a final F1 score of 0.79. Lai et al. Lai et al. (2019) developed a streamlined two-stream CNN with each stream containing only 8 layers. This model achieved a sensitivity of 89.5% and a specificity of 82.7% on the PhysioBank dataset (PhysioBank, 2000). The extracted cardiac rhythm features, specifically RR intervals and F-wave frequency spectra, served as dual inputs for the neural network. Similarly, Fan et al. Fan et al. (2018) developed a multi-scaled two-stream network with different filter sizes at each stream to capture features of different scales using single-lead ECGs from PhysioNet/Cinc 2017, achieving an F1 score of 0.8355. Phukan et al. Phukan et al. (2023) did a systematic experiment on selections of filter size, number of layers, and activation function on multiple standard datasets. They concluded that the best 5-layer CNN with activation function of exponential linear unit and kernel size 4 × 1 provides the highest accuracy of 99.84% for 5s ECG segments.
2) **Recurrent Neural Networks (RNNs):** An RNN is a type of neural network designed to effectively process sequential data by maintaining a memory of previous inputs, making it suitable for classifying time-series signals, e.g., AF detection. For example, Schwab et al. Schwab et al. (2017) built an ensemble of RNNs to jointly distinguish AF from normal ECGs, resulting in 0.79 of F1 score on the PhysioNet/Cinc 2017 dataset. Faust et al. Faust et al. (2018) utilized RNNs, specifically the long short-term memory (LSTM) architecture, to analyze ECGs from the MIT-BIH Atrial Fibrillation Database, achieving an accuracy rate of 99.77% for AF detection. Wang et al. Wang et al. (2023a) proposed a dual-path RNN which includes the intra- and inter-RNN modules to study the global and local aspects for end-to-end AF recognition. They used the PhysioNet/Cinc 2017 dataset to validate their model and achieved an F1 score of 0.842. More recently, bidirectional long short-term memory (Bi-LSTM), a type of RNN architecture capable of capturing both past and future context in sequential data, has been used to discern AF. Ramkumar et al. Ramkumar et al. (2022) created an auto-encoder and Bi-LSTM-based network to detect AF among others. This method integrated a reconstruction error from the auto-encoder into the total loss function, leading to a sensitivity of 92% and specificity of 97% on the PhysioNet/Cinc 2017 dataset.
3) **CNN-RNNs:** CNN-RNN hybrids combine the morphological feature extraction capabilities of 1D-CNNs with the temporal pattern recognition strengths of RNNs to address complex tasks such as AF detection from ECG signals. For example, Limam et al. Limam and Precioso, (2017) used dual CNNs to process the inputs consisting of both ECGs and heart rates independently, and then the processed features were merged into RNN to learn the temporal patterns, achieving a validated F1 score of 0.856 on the PhysioNet/CinC 2017 dataset. Wang et al. Wang and Li, (2020) combined CNN with Bi-LSTM, exploring two concatenation strategies: a parallel concatenation of CNN and Bi-LSTM, and a sequential one where the CNN output feeds into the Bi-LSTM. They evaluated the methods on the MIT-BIH dataset, reporting a final F1 score of 0.82 for the sequential strategy. Zhang et al. developed a model that merges a multi-branch CNN (MCNN) with Bi-LSTM to improve AF detection from short ECG recordings (Zhang et al., 2022). Unlike our multi-branching approach for addressing the imbalanced data issue, their model extracted features from various segments of a single-lead ECG, which were then processed by the Bi-LSTM. They tested the model on the PhysioNet/CinC 2017 dataset, achieving an F1 score of 0.7894.
4) **Attention-based networks:** The attention mechanism (Bahdanau et al., 2014; Vaswani et al., 2017) in deep learning dynamically weighs the importance of different input features, allowing models to focus more on relevant data while processing a task. This special capability can facilitate pattern recognition in ECG signals, enhancing the accuracy and efficiency of AF detection. For example, Gao et al. Gao et al. (2021) designed a residual-based temporal attention CNN, generating temporal informative features related to AF, so as to consider the semantic information to achieve better performance. This model achieved an accuracy of 85.43% on the PhysioNet/CinC 2017 dataset. Nankani et al. Nankani and Baruah, (2022) investigated the transformer network for AF detection and underscored clinically relevant signal timestamps triggering the diagnosis, achieving an F1 score of 0.87 on the PhysioNet/Cinc 2017 dataset. Rohr et al. Rohr et al. (2022) explored and assessed two advanced models for AF detection: a transformer-based DualNet architecture and a CNN-LSTM hybrid model, achieving F1 scores of 0.9127 and 0.9072, respectively, on the PhysioNet/CinC 2017 dataset.

As highlighted above (Andreotti et al., 2017; Fan et al., 2018; Lai et al., 2019; Phukan et al., 2023), 1D-CNNs have exhibited their effectiveness in identifying morphological features and comprehending temporal variations in time series data, demonstrating superior capability in AF detection using single-lead ECG signals. However, despite the promising utility of 1D-CNNs in time series analysis, comparative studies in the literature Ullah et al. (2021) and Wu et al. (2018) indicate that 1D-CNNs often yield lower prediction accuracies than their 2D counterparts under similar network configurations for ECG classification tasks.

This discrepancy can be attributed to the richer, more comprehensive information encapsulated in 2D input data, coupled with the inherently superior capacity of 2D CNNs for feature extraction and interpretation.

Owing to the outstanding performance and strong ability in pattern recognition, 2D CNN has been explored for ECG classification by virtue of its capacity to smartly suppress measurement noises and extract pertinent feature maps using convolutional and pooling layers (Huang et al., 2019). For example, Izci et al. Izci et al. (2019) engaged a 2D CNN model to investigate ECG signals for arrhythmia identification. They segmented the ECG signals by heartbeats and directly converted each heartbeat into grayscale images, which served as the input of the 2D CNN model. Similarly, Jun et al. Jun et al. (2018) proposed to combine 2D CNN and data augmentation with different image cropping techniques to classify 2D grayscale images of ECG beats. However, these end-to-end 2D CNNs are directly fed with original ECG beat segments without considering the possible noise contamination. Moreover, the 2D input data were created by directly plotting each ECG beat as a grayscale image with unavoided redundant information residing in the image background. This procedure requires extra storage space for training data and increases the computational burden without extracting relative features inherent in the ECG beats.

ECG signals generally consist of various frequency components, which can be used to identify disease-altered cardiac conditions. Wavelet transform (WT) (Daubechies, 1990; Yao et al., 2017; van Wyk et al., 2019) has been proven to be a useful technique for extracting critical time-frequency information pertinent to disease-altered ECG patterns (Kutlu and Kuntalp, 2012; He et al., 2018). As such, WT is favored as a feature-preprocessing procedure that converts 1D ECG signals into 2D images containing time-frequency features. The resulting 2D feature images then serve as the input of CNNs for ECG classification instead of the original 2D ECG plots. For instance, Xia et al. Xia et al. (2018) engaged the short-term Fourier transform (STFT) and stationary wavelet transform to convert ECG segments into 2D matrices which were then fed into a three-layer CNN for AF detection. Wang et al. Wang et al. (2021) combined the time-frequency features extracted by Continuous Wavelet Transform (CWT) and R-interval features to train a 2D CNN model for ECG signal classification. Wu et al. Wu et al. (2019) built a 2D CNN based on time-frequency features of short-time single-lead ECGs extracted from three methods, i.e., STFT, CWT, and pseudo Wigner-Ville distribution, to detect arrhythmias. Huang et al. Huang et al. (2019) developed an ECG classification model by transforming ECG signals into time-frequency spectrograms using STFT and feeding them into a three-layer 2D CNN. Li et al. Li et al. (2019) included three different types of wavelet transform (i.e., Morlet wavelet, Paul wavelet, Gaussian Derivative) to create 2D time-frequency images as the input data to the 2D CNN-based ECG classifier. The above literature unequivocally demonstrates that incorporating frequency information through the WT can significantly enhance the efficacy of ECG classification, underscoring the vital role of frequency domain analysis in AF identification.

In addition to effective information extraction from ECG time series, the realization of the full data potential is heavily reliant on advanced analytical models. Although the abovementioned works have validated the superiority of 2D CNN-based approaches, the shallow network structures with a limited number of layers can potentially hinder the extraction of deeper features. Naturally, the capacity for a neural network to learn is enhanced by an increase in the number of layers. However, having a deeper network structure can result in a gradient dissipation problem, which impedes convergence during network training, leading to suboptimal prediction performance. To cope with this issue, the residual neural network (ResNet) has been developed with an important modification, i.e., identity mapping, induced by the skip connection technique (He et al., 2016), which has wide applications in classifying the ECG signals. For example, Jing et al. Jing et al. (2021) developed an improved ResNet with 18 layers for single heartbeat classification. Park et al. Park et al. (2022) used a squeeze-and-excitation ResNet with 152 layers and compared the model performance trained by ECGs from a 12-lead ECG system and single-lead ECG data. Guan et al. Guan et al. (2022) proposed a hidden attention ResNet to capture the deep spatiotemporal features using 2D images converted from ECG signals.

Automated ECG classification also suffers from the long-standing issue of imbalanced data in machine learning. Diverse sampling and synthetic strategies have been proposed to address the imbalanced data issue, which focuses on creating a balanced training dataset out from the original imbalanced data to mitigatethe potential bias introduced by imbalanced data distribution during model training (He and Garcia, 2009). Frequently employed techniques consist of random over-sampling and under-sampling, informed adaptive undersampling, and synthetic minority over-sampling technique (SMOTE) (Gao et al., 2019; Wang and Yao, 2021; Qiu et al., 2022). For example, Luo et al. Luo et al. (2021) engaged SMOTE to synthesize minority samples and create a balanced training dataset for automated arrhythmia classification. Ramaraj et al. Ramaraj and Clement Virgeniya, (2021) incorporated an adaptive synthetic sampling process into the training of deep learning models built with gated recurrent units to address the class imbalance problem for ECG pattern recognition. Nurmaini et al. Nurmaini et al. (2020) compared sampling schemes of SMOTE and random oversampling with RNN and concluded that the balanced dataset created by SMOTE significantly improved the classification performance. In addition to fabricating balanced ECG datasets, Gao et al. Gao et al. (2019) and Petmezas et al. Petmezas et al. (2021) proposed to engage dynamically-scaled focal loss function to suppress the weight of loss corresponding to the majority class, so that their contribution to the total loss is reduced to alleviate the class imbalance problem. However, this method requires the preassumption of a focusing parameter to modulate the effect of the majority class on the total loss. Existing methods mainly focus on using sampling and synthetic strategies or modifying the loss function, little has been done to create new network structures without making extra assumptions and feature engineering to cope with the imbalanced data issue in AF identification from ECG signals.

## 3 Materials and methods

### 3.1 Dataset

In this study, two AF databases from different sources, i.e., ECG recordings from PhysioNet/CinC challenge 2017 (Goldberger et al., 2000; Clifford et al., 2017) and ECG PDFs from OUHSC, are used to evaluate the performance of data-driven detection methods. Both databases consist of short single-lead ECG recordings for AF and non-AF patients. PhysioNet/CinC Challenge 2017 is an open database including 8,528 single-lead ECG signals and their annotations. Among them, 5050 ECG recordings are labeled as normal sinus rhythm while 738 signals are annotated as AF. The sampling frequency of recordings is 300 Hz and the duration of ECG signals varies from 9s to 30s. The OUHSC database contains ECG signals in PDF format with 33 recordings from AF subjects and 227 normal samples, which are annotated by cardiologists from OUHSC. Each recording has a duration of around 30s with a sampling frequency of 60 Hz.

### 3.2 ECG signal preprocessing

**FIGURE 1:**
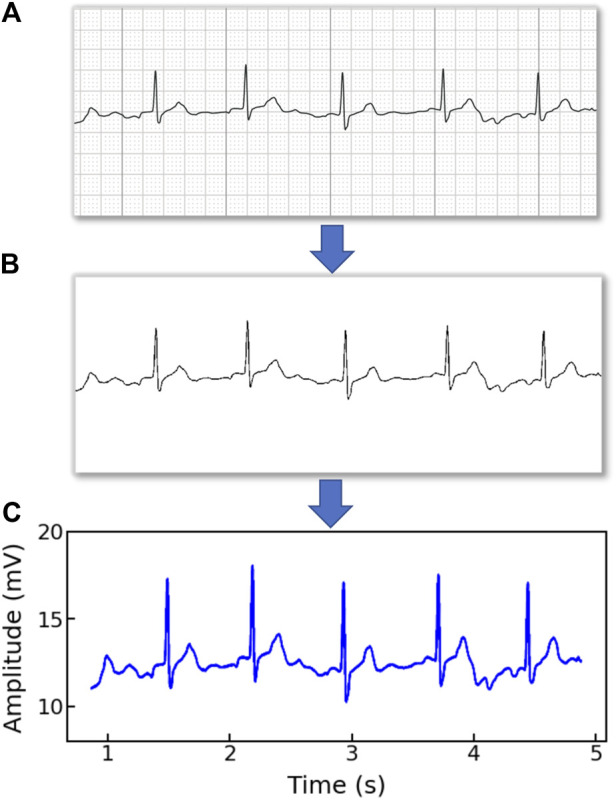
An example of **(A)** a raw image recording of an ECG segment in PDF format, **(B)** the ECG image that filters out the grid background, **(C)** the digitalized ECG time series signal.

Note that the original ECG recordings from OUHSC are in PDF format, as shown in Figure 1A. It is necessary to accurately extract the numerical ECG readings from the PDF files for further data preprocessing and analysis, which is achieved by the following procedure:

•*Transforming PDF files into gray-scale images represented by 2D-pixel matrices*: We discretize the 2D image into a pixel matrix. Then, each pixel is converted to a fixed number of bits to represent the gray-scale intensity of the corresponding point in the image. As shown in Figure 1A, the ECG signals are displayed in the darkest color on the plot with the color intensity of 1, i.e., *h*(*m*, *n*) = 1, while the grid lines appear in a lighter color, i.e., 0 < *h*(*m*, *n*) < 1, where *h*(*m*, *n*) denotes the color intensity of the pixel at column *m* and row *n*. Note that the background color intensity is 0.
•*Removing grid lines from the ECG plot*: We replace the pixel shade values of the grid lines with the background color value: i.e., *h*(*m*, *n*∣*h*(*m*, *n*) < 1) = 0. This allows the ECG signals to distinguishably stand out, as illustrated in Figure 1B. The quantized image is thus encoded into a binary digital format, i.e., black as “1” and white as “0”. As such, the entire ECG image is transformed into a binary digital matrix without the grid lines.
•*Extracting the digital ECG time series*: The positions of black pixels (i.e., ECG signal) in the binary matrix are further extracted, which are represented as a set of (*m*, *n*) pairs:

$$S=\left\{\left(m,n\right)|h\left(m,n\right)=1\right\}$$

The resulting *S* is then used to reconstruct the digital ECG time series, where *m* stands for the time course, and *n* corresponds to the magnitude of the ECG signal. As such, we are able to extract the ECG recordings from the PDFs to digitalized ECG time series signals (Figure 1C), which will be used for further processing and model training.

Raw ECG recordings are often contaminated by noises, such as baseline wandering, electromyography disturbance, and power-line interference (Mian Qaisar, 2020), which will negatively impact the information extraction and model performance. In this work, we engage BioSPPy, a toolbox for biosignal processing written in Python, for ECG signal denoising. The BioSPPy library provides comprehensive functions for processing ECG signals including functions for importing ECGs, filtering out interfering components, and correcting baseline wandering (PIA-Group, 2021). Specifically, after loading the ECG data, we apply a high-pass filter to remove the low-frequency noise (e.g., baseline wandering), a notch filter to remove power-line interference, and a low-pass filter to filter out the high-frequency noise.

### 3.3 Continuous wavelet transform

ECG signals encompass multiple feature components in both the time and frequency domains. In this study, we engage the continuous wavelet transform (CWT) to extract time-frequency features from ECGs due to its excellent performance in the analysis of transient and non-stationary time series signals (Keissar et al., 2009). CWT is the most popular tool for time-frequency analysis that reflects the frequency components of data changing with time. CWT is verified to outperform the traditional STFT due to its ability to provide multi-resolution decompositions of the signal, which allows for a trade-off between time and frequency resolution, i.e., higher frequency resolution for signals with sharp transients and higher time resolution for signals with slow-varying frequency content (Dokur and Ölmez, 2001). Additionally, compared to discrete wavelet transform (DWT), CWT remedies non-stationarity and coarse time-frequency resolution defects and supports the extraction of arbitrarily high-resolution features in the time-frequency domain (Addison, 2005).

The CWT of the ECG time-series signal denoted as *x*(*t*) is achieved according to:

$$T\left(a,b\right)=\frac{1}{\sqrt{a}}\int_{-\infty }^{+\infty }x\left(t\right)\psi \left(\frac{t-b}{a}\right)dt$$ (1)

where *T*(*a*, *b*) stands for the intensity of transformed signals, *ψ*(⋅) is the wavelet basis (also known as the mother wavelet), *a* is the scale factor quantifying the compressed or stretched degree of a wavelet, and *b* is the time shift parameter defining the location of the wavelet. The scale can be used to derive the characteristic frequency of the wavelet as (Wu et al., 2019):

$$F=\frac{F_{c}\times f_{s}}{a}$$ (2)

where *F*_*c*_ is the center frequency of the mother wavelet and *f*_*s*_ is the sampling frequency of the signal. This relationship shows that smaller (larger) values of *a* correspond to higher (lower) frequency components. In CWT, the mother wavelet plays a critical role in time-frequency analysis, the choice of which depends on its similarity with the original signal (Ngui et al., 2013). Here, the Mexican hat wavelet (mexh) is chosen to serve as the mother wavelet because its shape is similar to the QRS waves and it is commonly used in ECG signal analysis (Wang et al., 2021). Specifically, the mexh is the second derivative of a Gaussian function (Addison, 2005), which is defined as:

$$\psi \left(t\right)=\frac{2}{\sqrt{3}\sqrt[{4}]{\pi }}\exp\left(-\frac{t^{2}}{2}\right)\left(1-t^{2}\right)$$ (3)

Continuously changing the scale factor *a* and time shift parameter *b* generates the 2D wavelet coefficients *T*(*a*, *b*), which can be viewed as a 2D scalogram of the ECG signal in both the time and frequency domain (Wang et al., 2021).

**FIGURE 2:**
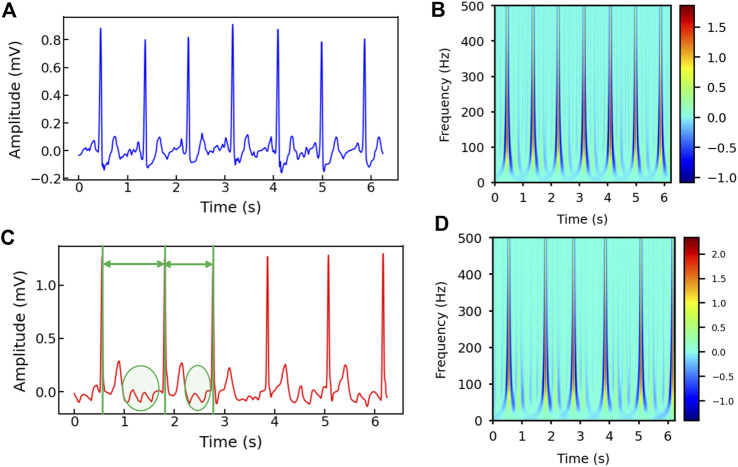
**(A)** The raw ECG signal from Physionet labeled as normal and **(B)** its corresponding 2D CWT scalogram. **(C)** The raw ECG signal from Physionet labeled as AF and **(D)** its corresponding 2D CWT scalogram. Note that the RR intervals are different in the AF sample and irregular F waves (circled) appear in **(C)**.

Figures 2A–D show the healthy and AF examples of the raw ECG signals obtained from PhysioNet and their 2D time-frequency patterns after CWT transformation with mexh wavelet, respectively. The colors in the scalogram indicate the energy density of the signal component at the corresponding frequency and time (Addison, 2005; He et al., 2018). According to Figure 2A,C, two general differences can be observed: 1) The AF ECG signal lacks a distinct P wave, while it shows a fast and chaotic F wave due to the atrial fluttering (Figure 2C), in comparison to a normal ECG signal (Figure 2A); 2) Irregular RR intervals are observed in AF ECG (Figure 2C) caused by a non-synchronized ventricular response to the abnormal atrial excitation (He et al., 2018). The discriminative information in the time domain can also be captured by the CWT scalograms shown in Figures 2B,D. By using a 2D CNN to analyze the visual representation of 2D time-frequency scalograms, we can better understand the features that distinguish AF from normal heart rhythms and make more accurate predictions.

### 3.4 Convolutional neural network

We engage CNN to build a data-driven classifier for differentiating AF samples from normal ECG samples. CNN is a type of network architecture specifically designed to process data that has a grid-like structure such as images (Khan et al., 2020). As opposed to traditional multilayer perceptron networks (MLPs), where the input of each neuron consists of the outputs of all the neurons from the previous layer, the neuron in CNN only receives its input from a localized region of the previous layer, known as its receptive field. The main building blocks of a CNN are convolutional layers, pooling layers, and fully connected layers.

Convolutional layers are responsible for performing a convolution operation on the input data, using a set of filters to extract local features in the data, and producing a feature map that summarizes such local information. Let *θ* and *X* denote the filter (also known as the kernel) and the input. The convolution operation works as follows:

$${\left(X⊗\theta \right)}_{ij}=\overset{s_{1}-1}{\underset{m=0}{\sum }}\overset{s_{2}-1}{\underset{n=0}{\sum }}X\left(i+m,j+n\right)\theta \left(m,n\right)$$ (4)

where *s*_1_ and *s*_2_ denote the size of the 2D kernel, and (*i*, *j*) denotes the location on the 2D input (e.g., image). After being applied with the activation function, the feature map of the input is obtained as (LeCun and Bengio, 1995; Jing et al., 2021):

$$X_{q}^{l}=\sigma \left(\underset{p}{\sum }\theta_{pq}^{l}⊗X_{p}^{l-1}+b_{q}^{l}\right)$$ (5)

where $X_{q}^{l}$ is the *q*th feature at layer *l*, $X_{p}^{l-1}$ is the *p*th input feature map of the previous (*l* − 1)-th layer, *σ* denotes the activation function to induce the non-linearity in the functional mapping, and *b*_*q*_ represents the bias. This procedure is repeated by applying multiple filters to generate multiple feature maps to capture different characteristics of the input. Note that kernels are shared across all the input positions, which is also called weight sharing, the key feature of CNN. The weight-sharing technique guarantees the extracted local patterns are translation invariant and increases computational efficiency by reducing the model parameters to learn compared with fully connected neural networks.

The pooling layer mimics the human visual system by combining the outputs of multiple neurons (i.e., clusters) into a single neuron in the next layer, effectively creating a condensed representation of the input. The pooling significantly reduces the spatial resolution and only focuses on the prominent patterns of the feature maps, making the network more robust to small translations and distortion in the input data (Xia et al., 2018). Popular pooling techniques include maximum pooling, average pooling, stochastic pooling, and adaptive pooling. They are typically performed on the values in a sub-region of the feature map (Akhtar and Ragavendran, 2020).

The fully-connected layers form a dense network that can learn complex non-linear relationships between the inputs and outputs. It takes the output of the previous layer, which is typically a high-dimensional tensor containing discriminant features extracted by convolutional and pooling layers, and flattens it into a one-dimensional vector. This vector is then used as the input to a fully connected layer. The fully-connected layer is similar to an MLP in that every neuron in one layer is connected to every neuron in the next layer. By using a proper activation function, the neural network is able to produce classification decisions (Nurmaini et al., 2020). By stacking these building blocks (convolutional layers, pooling layers, and fully connected layers) in various combinations, CNN is able to learn complex features in the input data, allowing them to effectively solve a wide range of image and signal processing tasks (Andreotti et al., 2017).

### 3.5 2D CNN with ResNet

We propose to engage 2D CNN to investigate the 2D time-frequency scalograms converted from denoised ECG signals by CWT for AF identification. It has been demonstrated that the substantial depth of the convolutional network is beneficial to the network performance (Simonyan and Zisserman, 2014). However, as the number of convolutional layers increases, the training loss stops further decreasing and becomes saturated because of the gradient dissipation issue. As such, a CNN with a deeper architecture, counterintuitively, sometimes incurs a larger training error compared to its shallow counterpart upon convergence (He et al., 2016). To solve such network degradation and gradient vanishing problems, the residual network (ResNet) has been developed to improve the accuracy of CNNs with considerably increased depth.

**FIGURE 3:**
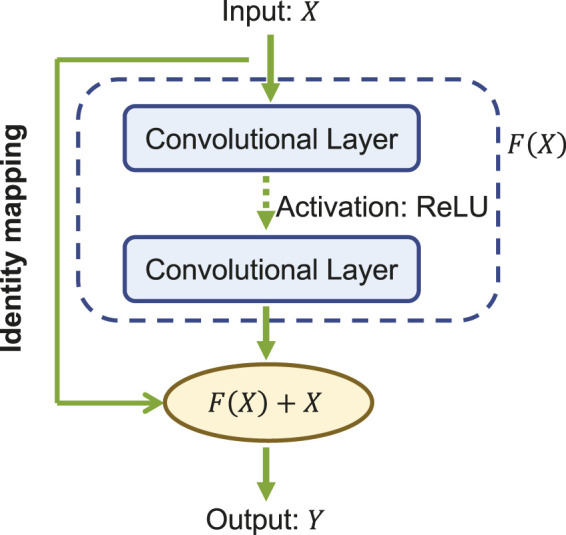
A building block of the ResNet.

The core of ResNet is the residual learning technique (He et al., 2016). Specifically, instead of using the stacked convolutional layers to directly fit the underlying mapping from the input to the output, ResNet focuses on fitting a residual mapping. Figure 3 shows a ResNet building block with input *X* and its corresponding output mapping *Y*. The residual block engages a shortcut connection that bypasses one or more convolutional layers and allows the information to flow directly from the input to the output. As such, the input *X* is added to the output of the block *F*(*X*) (enclosed by the dashed circle in Figure 3, allowing the network to learn the residual mapping represented as *Y* = *F*(*X*) + *X* instead of learning the direct mapping as *Y* = *F*(*X*). This design mitigates the gradient vanishing problem and allows for deeper networks to be trained effectively.

**FIGURE 4:**
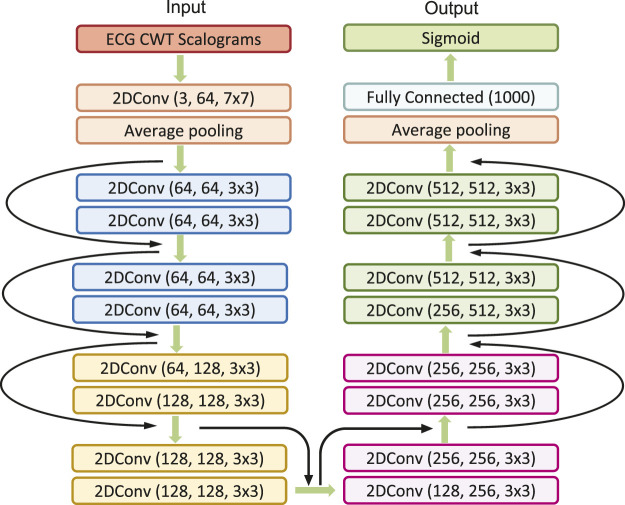
The detailed architecture of ResNet18.

In our study, we engage the ResNet with 18 layers (ResNet18) to build the AF classifier because ResNet18 has been proven to be able to generate a comparable result with a faster convergence compared to a deeper counterpart (He et al., 2016). Figure 4 shows the detailed structure of ResNet18. Note that the notation of 2DConv(*n*_*input*_, *n*_*output*_, *n*_*fdim1*_ × *n*_*fdim2*_) denotes that, in the current 2D convolutional layer, there are *n*_*input*_ input channels, *n*_*output*_ output channels (i.e., number of filters) with the 2D filter size of *n*_*fdim1*_ × *n*_*fdim2*_. For example, (64, 128, 3 × 3) indicates that this convolutional layer is composed of 128 filters with the filter size of 3 × 3 applied on the input data with 64 channels.

### 3.6 Multi-branching convolutional network

**FIGURE 5:**
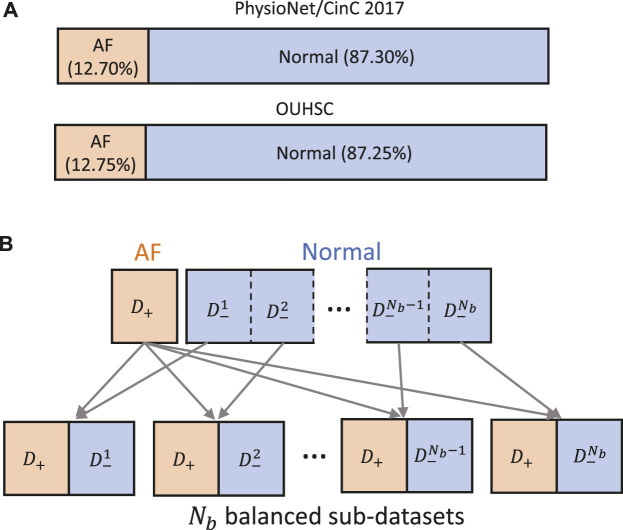
**(A)** Class distribution in PhysioNet/Cinc 2017 and OUHSC datasets. **(B)** Illustration of creating *N*_*b*_ balanced sub-datasets to train our MB-ResNet model.

**FIGURE 6:**
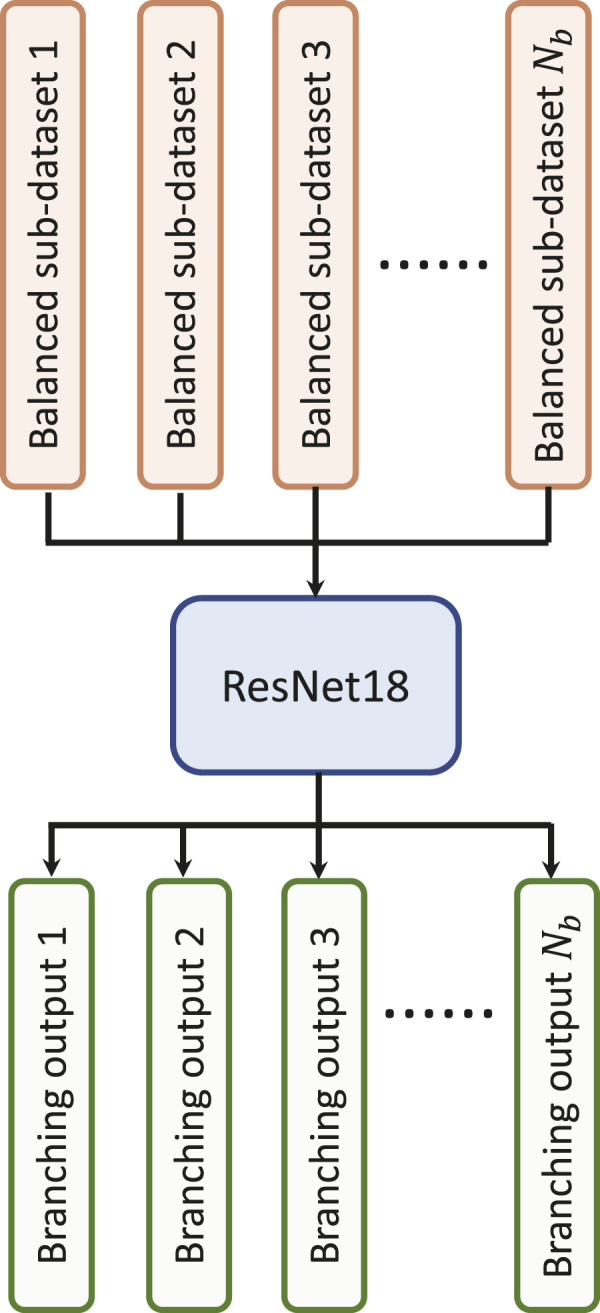
Illustration of the multi-branching architecture.

Data-driven identification of AF from ECG recordings generally suffers from imbalanced data issues. Figure 5A presents the distribution of AF and normal samples in Physionet/CinC 2017 and OUHSC datasets, illustrating a normal to AF sample ratio of approximately 7:1 for both. To address the data imbalance issue, we create *N*_*b*_ balanced datasets from the original data *D* = {*D*_−_, *D*_+_}, where *D*_−_ denotes the majority normal ECG samples and *D*_+_ stands for the minorityset, i.e., the entire AF training samples. *D*_−_ is partitioned into multiple subsets $D_{-}=\cup_{i=1}^{N_{b}}D_{-}^{i}$ , where each subset *D*_*i*_ is roughly equivalent in size to *D*_+_. The normal subsets *D*_*i*_ for *i* = 1, . . ., *N*_*b*_ are then paired with *D*_+_ to formulate balanced sub-datasets. Each balanced subset, denoted as $D_{i}=\left\{D_{-}^{i},D^{+}\right\}$ for *i* = 1, . . ., *N*_*b*_, is processed through the ResNet core, with individual branches trained on their respective balanced sub-datasets. Figure 5B visualizes this method of partitioning the original dataset D into *N*_*b*_ balanced sub-datasets, i.e., *D*_*i*_ for *i* = 1, . . ., *N*_*b*_, which serve as the balanced input in Figure 6. This strategic partitioning and training approach ensures a comprehensive model learning from a balanced representation of AF and normal ECG samples (Wang and Yao, 2021; Wang et al., 2022; Wang et al., 2023b).

In the current investigation, we aim to identify AF samples from normal ECG samples. The neural network is expected to produce high probabilities (close to 1) for AF samples and low probabilities (close to 0) for normal ECG samples. We choose the binary cross-entropy as the loss function for MB-ResNet, which is defined as:

$$\begin{array}{ll} L\left(\omega ;D\right) & =-\overset{N_{d}}{\underset{j=1}{\sum }}\overset{N_{b}}{\underset{i=1}{\sum }}I\left(\;j\in D_{i}\right)\left(y_{j}\log\left({\hat{P}}_{i}\left(\omega ;X^{j}\right)\right)\right.\\ & \;\left.+\left(1-y_{j}\right)\log\left(1-{\hat{P}}_{i}\left(\omega ;X^{j}\right)\right)\right) \end{array}$$ (6)

where ***ω*** denotes the neural network parameter set, *X* ^*j*^ and *y*_*j*_ stand for one input sample and its corresponding true label respectively, I(⋅) denotes the indicator function, *N*_*d*_ is the total number of the training samples, and ${\hat{P}}_{i}\left(\omega ;X^{j}\right)$ represents the predicted probability for AF at the *i*th branching output given the input signal *X* ^*j*^.

The adaptive momentum method (Adam) (Kingma and Ba, 2014) is adopted to minimize the loss function and update the network parameters. In the inference stage, the MB network generates *N*_*b*_ predictions for AF probability, which correspond to the *N*_*b*_ branching outputs. The final predicted probability for AF $\left(\hat{P}\right)$ is determined by taking the average of the *N*_*b*_ outputs:

$$\hat{P}=\frac{1}{N_{b}}\overset{N_{b}}{\underset{i=1}{\sum }}{\hat{P}}_{i}$$

where ${\hat{P}}_{i}$ is the predicted probability of *i*th branching output.

## 4 Experimental design and results

### 4.1 Experimental design

We validate and evaluate the performance of the proposed CWT-MB-ResNet framework using both OUHSC and Physionet Challenge datasets. In this study, the training and testing datasets are split interpatiently for both data sources. This ensures that no overlap exists between the patients in the training set and those in the testing set. We allocate 80% of the total samples for the training purpose and the remaining 20% for testing, applied on both datasets.

**FIGURE 7:**
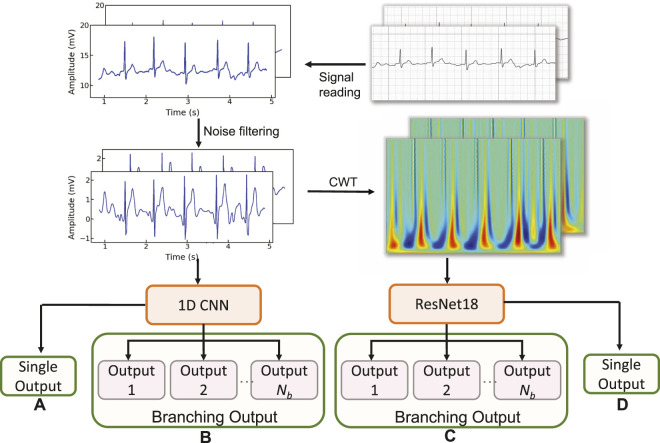
The flowchart of the experimental design: **(A)** 1D-CNN; **(B)** 1D-MB-CNN; **(C)** CWT-MB-ResNet; **(D)** CWT-ResNet.

**FIGURE 8:**
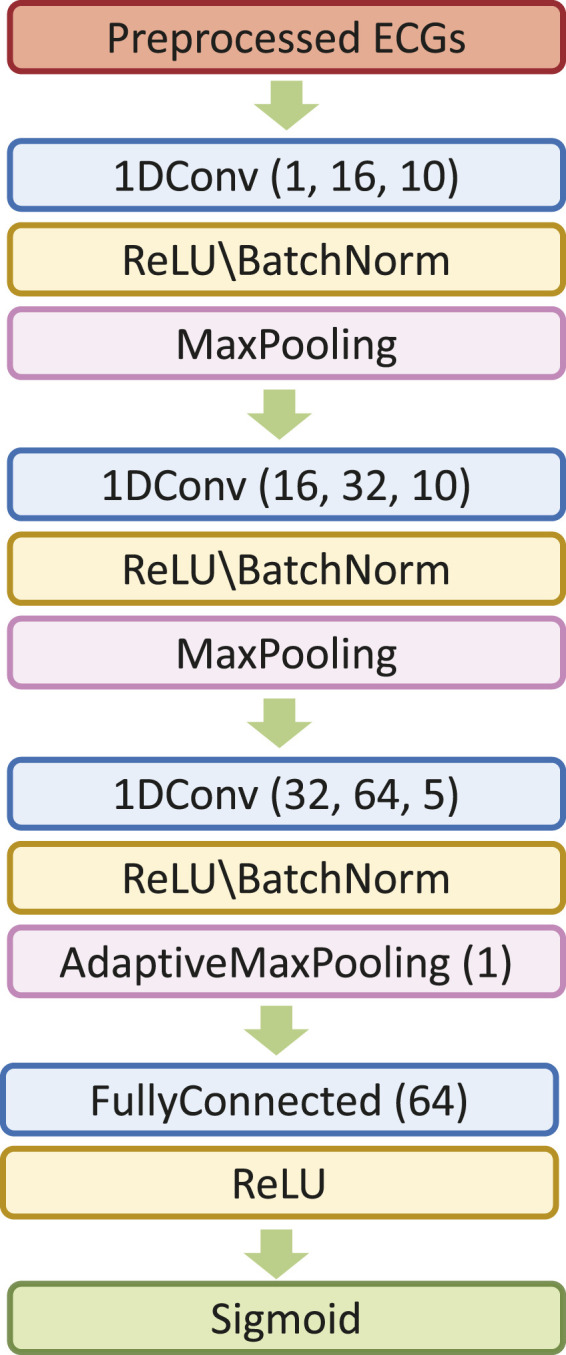
The 1D-CNN architecture.

We first explore the impact of the learning rate on the training outcomes of the proposed CWT-MB-ResNet. We then conducted a comparison study to showcase the significance of ECG digitalization for the proposed multi-branching ResNet (MB-ResNet) model in identifying the AF samples. Next, we compare the performance of our CWT-MB-ResNet with 1D-CNN (Figure 7A), 1D-CNN with the multi-branching network (1D-MB-CNN) (Figure 7B), and ResNet with CWT features (CWT-ResNet). Note that the input of 1D-CNN and 1D-MB-CNN consists of the denoised ECG time series. The detailed 1D-CNN architecture is illustrated in Figure 8, including three convolutional layers followed by pooling layers to reduce the dimensionality of the data, a batch-normalization layer to stabilize the network training, and one fully connected layer to make the final prediction. Note that the notation of 1DConv(*n*_*input*_, *n*_*output*_, *n*_*fdim*_) indicates that, in the current 1D convolutional layer, there are *n*_*input*_ input channels and *n*_*output*_ output channels (i.e., number of filters) with a 1D filter size of *n*_*fdim*_.

The classification performance will be evaluated with three metrics: Receiver-Operating-Characteristic (ROC) Curve, Precision-Recall (PR) Curve, and F1 score, which will be calculated using the test set. The ROC provides the graphic representation of the trade-off between the true positive rate (TPR) and the false positive rate (FPR) for different threshold settings. The area under ROC (AUROC) is often used as a metric to compare different models, with a larger AUROC indicating a better-performing classifier. A good model typically has a ROC curve that is situated toward the top-left corner of the graph. The PRC illustrates the interplay between a predictive model’s precision and recall metrics across a range of probability thresholds. A good classifier has the PR curve towards the top-right corner. A higher area under PRC (AUPRC) value suggests a more effective model. The F1 score quantifies the equilibrium between a model’s precision and recall for a binary classifier system by computing their harmonic mean, which is defined as

$$F1=\frac{2\times Precision\times Recall}{Precision+Recall}$$

Note that the F1 score ranges from 0 to 1, where a score of 1 indicates a perfect balance between precision and recall and a good overall prediction performance.

### 4.2 The effect of the learning rate on CWT-MB-ResNet

In this study, we initiate the analysis by transforming ECG time series data into 2D scalograms utilizing CWT. These scalograms encapsulating both time and frequency information are input into our tailored MB-ResNet model. Specifically, we employ ResNet18 due to its proven efficacy in achieving results comparable to those of its deeper counterparts, while also ensuring faster convergence rates (He et al., 2016). The architecture of ResNet18, as adopted from He et al. (2016) and illustrated in Figure 4, comes with a predefined set of network architecture parameters, including number of layers, kernel size, and number of residual blocks.

In addition to selecting ResNet18 for its balance between efficiency and performance, the learning rate has a critical influence on the training outcomes. To further optimize our model, we conducted an experiment specifically focused on assessing the impact of various learning rates on the model’s performance, particularly looking at the F1 score on the test set across both datasets used in our study. Table 1 summarizes the performance of the MB-ResNet given different learning rates. For both datasets, the highest F1 score achieved is 0.8865 for PhysioNet/CinC 2017 and 0.7396 for OUHSC datasets when the learning rate is set as 0.001. This indicates that a learning rate of 0.001 is the most effective in training our MB-ResNet model.

**TABLE 1:** F1 scores on the testing set given different learning rates for MB-ResNet training.

learning rate	0.0001	0.0005	0.001	0.005	0.01
F1 (PhysioNet)	0.8493	0.8759	0.8865	0.8652	0.8580
F1 (OUHSC)	0.6854	0.7273	0.7396	0.7385	0.7151

### 4.3 The effect of ECG digitalization from PDFs on CWT-MB-ResNet

**FIGURE 9:**
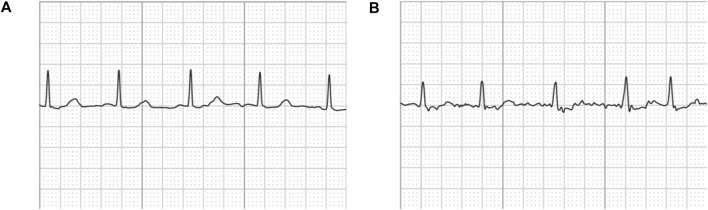
Examples of ECG segments: **(A)** normal sample; **(B)** AF sample.

We carry out a comparative analysis to demonstrate the importance of digitizing ECG records from their original PDF format. Specifically, we transform the original ECG PDFs into image files (i.e., Portable Network Graphic (.PNG) files) and apply segmentation to augment the sample sizes. Figure 9 illustrates examples of the resulting ECG images from normal and AF categories, which directly serve as inputs for our MB-ResNet without further preprocessing.

**FIGURE 10:**
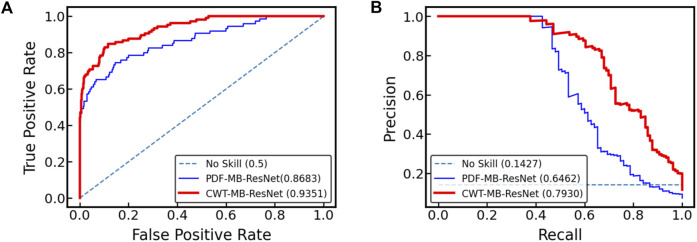
Comparison of **(A)** ROC and **(B)** PR curves for the MB-ResNet model trained with two different data preparation techniques: one involving 2D scalograms derived from digitalized ECGs which are denoised and processed through CWT (CWT-MB-ResNet), and the other using unprocessed ECG images directly from raw PDF files (PDF-MB-ResNet).

Figure 10 displays the ROC and PR curves generated by two variants of the MB-ResNet model: one trained on 2D scalograms derived from digitalized ECGs after undergoing denoising and CWT (referred to as CWT-MB-ResNet), and the other trained on pure ECG images converted directly from raw PDF files (denoted as PDF-MB-ResNet). Utilizing the same MB-ResNet model, we observed a substantial increase in the area under both ROC and PR curves when the model inputs were 2D scalograms processed from digitalized ECGs compared with using raw ECG images directly. Specifically, our CWT-MB-ResNet model demonstrates superior performance with an AUROC of 0.9351, AUPRC of 0.7930, and an F1 score of 0.7396. This performance significantly surpasses that of the PDF-MB-ResNet trained by raw ECG images with an AUROC of 0.8683, AUPRC of 0.6462, and an F1 score of 0.6257, highlighting the efficacy of our digitalization and preprocessing procedure. The enhanced performance of the MB-ResNet model trained with 2D scalograms from digitalized ECGs, as compared to training with raw ECG images, is be attributed to several factors:

•The 2D scalograms provide a rich representation of temporal and frequency features, offering a more comprehensive dataset for the model to learn from.
•The raw ECG segmentation images contain large blank areas devoid of any ECG-related information, which do not contribute to learning discriminative features.
•The superimposed gridlines in the area could introduce noise into the data, potentially hindering the model’s training efficiency.

By training with 2D scalograms, the abovementioned issues are mitigated, allowing the MB-ResNet to focus on more relevant ECG features, leading to significant improvement in overall model performance.

### 4.4 Experimental results from the OUHSC dataset

**FIGURE 11:**
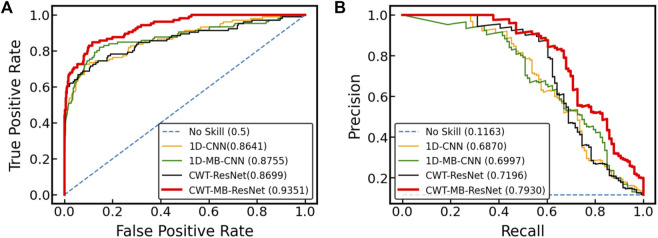
The comparison of **(A)** ROC and **(B)** PRC among different models using the OUHSC data.

Figure 11 displays the ROC and PR curves of all four models using the OUHSC dataset. The 2D ResNet models (i.e., CWT-ResNet and CWT-MB-ResNet), which use 2D scalograms transformed from ECG signals as the input, produce a larger area under the curves (both ROC and PR) compared to their 1D counterparts (i.e., 1D-CNN and 1D-MB-CNN). This demonstrates the efficacy of using the CWT to extract time-frequency features in the ECG signal analysis. Additionally, the models with an MB architecture (i.e., 1D-MB-CNN and CWT-MB-ResNet) produce a larger AUROC and AUPRC compared to models without MB outputs (i.e., 1D-CNN and CWT-ResNet), which highlights the effectiveness of using the MB structure in addressing imbalanced data issues. The ROC and PR plots demonstrate the superiority and robustness of the proposed CWT-MB-ResNet framework for identifying the AF samples.

Table 2 shows AUROC, AUPRC, and F1 scores generated from the four methods using the OUHSC dataset. The proposed CWT-MB-ResNet method generates the best AUROC, AUPRC, and F1 scores with values of 93.51%, 79.30%, and 0.7396. Note that the MB technique demonstrates its effectiveness on both 1D-CNN and CWT-ResNet as the AUROC, AUPRC, and F1 scores provided by the MB-based neural network models are higher than their non-MB counterparts. Moreover, the AF classifier using 2D-CNN-based ResNet18 supported by the time-frequency transformation of ECG time series presents a more potent predictive power than time sequence classification using 1D CNN. For example, CWT-MB-ResNet improves the AUROC, AUPRC, and F1 scores from 87.55%, 69.97%, and 0.6384% to 93.51%, 79.30%, and 0.7396 respectively compared with the 1D-MB-CNN.

**TABLE 2:** The comparison of AUROC, AUPRC, and F1 scores generated from 1D-CNN, 1D-MB-CNN, CWT-ResNet, and the proposed CWT-MB-ResNet using OUHSC data.

	1D-CNN	1D-MB-CNN	CWT-ResNet	CWT-MB-ResNet
AUROC	86.41%	87.55%	86.99%	93.51%
AUPRC	68.70%	69.97%	71.96%	79.30%
F1	0.6370	0.6384	0.7150	0.7396

### 4.5 Experimental results from the Physionet/CinC 2017 challenge dataset

**FIGURE 12:**
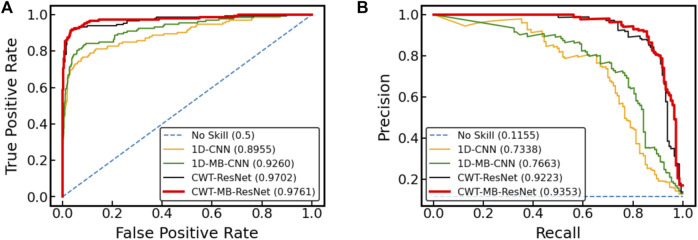
The comparison of **(A)** ROC and **(B)** PRC between different models using data from Physionet/Cinc 2017 challenge.

Figure 12 further shows the ROC and PRC analysis for the Physionet/Cinc 2017 challenge dataset. Similar to the results from the OUHSC dataset, the 2D ResNet models (CWT-ResNet and CWT-MB-ResNet) outperform their 1D counterparts (1D-CNN and 1D-MB-CNN) in both the ROC and PR spaces. Furthermore, the MB-based models (1D-MB-CNN and CWT-MB-ResNet) effectively account for the imbalanced data issues, exhibiting better performance compared to the non-MB-based models (1D-CNN and CWT-ResNet). Table 3 demonstrates the comparison of AUROC, AUPRC, and F1 scores provided by 1D-CNN, 1D-MB-CNN, CWT-ResNet, and CWT-MB-ResNet. Our CWT-MB-ResNet yields the best classification performance among the four methods, generating the highest AUROC, AUPRC, and F1 scores of 97.41%, 93.53%, and 0.8865. Especially, our CWT-MB-ResNet model improves the F1 score by 46.2% percent compared to the pure 1D-CNN with no CWT transform or MB structure.

**TABLE 3:** The comparison of AUROC, AUPRC, and F1 scores generated from 1D-CNN, 1D-MB-CNN, CWT-ResNet, and the proposed CWT-MB-ResNet using data from Physionet/CinC 2017 challenge.

	1D-CNN	1D-MB-CNN	CWT-ResNet	CWT-MB-ResNet
AUROC	89.55%	92.60%	97.02%	97.61%
AUPRC	73.38%	76.63%	92.23%	93.53%
F1	0.7219	0.7380	0.8690	0.8865

## 5 Discussion

### 5.1 Strengths of the proposed pipeline

This paper proposes a pipeline of CWT-MB-ResNet to identify the AF condition. The unique strengths of the proposed framework are:

1) **Digitalization of ECG readings in PDF:** This pipeline designed an ECG preprocessing method that can automatically convert ECG PDFs into digitalized, ready-to-use ECG time series data. This step is crucial for integrating machine learning models into clinical workflows, where ECGs are often archived in non-digitalized formats.
2) **Effectiveness of CWT representation:** The integration of CWT enhances feature extraction, enabling the model to better identify AF characteristics that might be missed by directly learning from raw time-series analysis alone. The resulted 2D ECG scalograms offer a rich representation of ECG data by encapsulating both time series and frequency components. The CWT-based feature reformulation can significantly enhance the model’s performance by providing more comprehensive information for classifying ECG signals.
3) **Advantage of the network design:** The use of ResNet18 as the foundation allows our model to benefit from the strengths in deep residual learning, enabling it to learn from significantly deepened convolutional layers with improved accuracy. The ResNet18 has demonstrated comparable results to its deeper counterparts, meanwhile keeping its computational efficiency. This is further enhanced by our innovative multi-branching design, which addresses the class imbalance issue by training each branch on a balanced subset of the original dataset while the core network is exposed to the entire range of samples. This approach ensures that both AF and normal class is adequately represented and learned during the training process, significantly enhancing the network’s ability to generalize across the imbalanced classes.

### 5.2 Discussion on the limitations

The proposed CWT-MB-ResNet framework, while effective, is not devoid of limitations. In our study, ECG segments were around 5 s long. However, analyzing longer ECG recordings will significantly increase computational complexity. This is due to the CWT method of processing data across both time and frequency domains at various scales, demanding more computational resources. Additionally, while our method effectively addresses class imbalance, its performance remains influenced by the quality and diversity of the training data, which is a long-lasting limitation of most data-driven machine learning models. This is evident from the differing performances on the PhysioNet and OUHSC datasets. Specifically, PhysioNet, with its larger and more diverse pool of 5,788 subjects, provides a richer training environment compared to OUHSC, which is limited to ECG samples from only 260 subjects. Despite utilizing segmentation to expand the sample size of the OUHSC dataset to 5,809, notable differences in performance metrics remain, as detailed in Tables 2 and 3. This suggests that merely increasing the sample size by segmentation cannot fully address the limitations posed by data diversity and quality. Additionally, deep learning models, including the proposed CWT-MB-ResNet, are often criticized for their “black box” nature. This means that while those models can make accurate predictions, the reasoning behind the predictions is not always clear or understandable to humans. This lack of interpretability can be a significant hurdle in clinical settings, making clinicians less confident in implementing machine learning models for automated diagnosis. One of our future research directions will focus on the development of interpretable models for AF detection.

### 5.3 Comparison with existing work

The direct comparison of our results with the values of performance metrics reported in other studies mentioned in Section 2 is neither fair nor feasible due to several factors: 1) variations in ECG duration used for training/testing data; 2) employment of non-unified metrics for evaluating model performance across studies; 3) variations in the proportions of training/testing data splits; 4) the model implementation on different databases. To enable a fairer and more meaningful comparison, we applied the ECG data from both the PhysioNet/CinC 2017 database and OUHSC to four deep learning models reviewed in Section 2, ensuring that the comparison is based on consistent data and preprocessing steps.

Table 4 summarizes the comparison results in terms of F1 score. Even though the proposed CWT-MB-ResNet model does not resort to complex neural network designs, it demonstrates the best F1 score compared with the other network structures developed in Andreotti et al. (2017); Limam and Precioso, (2017); Wang and Li, (2020); Gao et al. (2021). Specifically, the utilization of CWT distills both frequency and temporal insights from ECG signals, converting them into an image data format that significantly enriches the input information. We integrate the widely recognized image model, ResNet18 to achieve a robust interpretation of image data and meanwhile circumvent the gradient vanishing problem. Furthermore, the multi-branching structure is meticulously designed to address issues of data imbalance, ensuring that our model remains sensitive and accurate for both normal and AF classes.

**TABLE 4:** The comparison of F1 scores between the proposed CWT-MB-ResNet method with existing literature using data from Physionet/CinC 2017 and OUHSC.

Authors	Methods	F1 (PhysioNet)	F1 (OUHSC)
Andreotti et al. Andreotti et al. (2017)	ResNet	0.8405	0.7054
Limam et al. Limam and Precioso, (2017)	CRNN	0.8310	0.7323
Wang et al. Wang and Li, (2020)	CNN-Bi-LSTM	0.7094	0.6996
Gao et al. Gao et al. (2021)	Residual-based temporal attention	0.8172	0.7368
This paper	CWT-MB-ResNet	0.8865	0.7396

## 6 Conclusion

In this paper, we develop a novel framework based on Continous Wavelet Transform (CWT) and multi-branching ResNet for AF identification. We first transform the 1D ECG time series into 2D time-frequency scalograms to take into account various frequency components, which can serve as the input to the 2D CNN-based classifier. Second, we leverage the ResNet architecture to cope with the gradient dissipation problems in deep 2D CNN and increase the effectiveness of network training. Moreover, a multi-branching architecture is incorporated into the ResNet to mitigate the possible prediction bias caused by the imbalanced data issue. Finally, we implement the proposed CWT-MB-ResNet to predict AF using the ECG recordings from PhysioNet/CinC Challenge 2017 and the ECG PDFs from OUHSC. Experimental results show that the proposed CWT-MB-ResNet achieves the best prediction performance for both datasets in AF detection. The CWT-MB-ResNet framework has great potential to be applied in clinical practice to improve the accuracy in ECG-based diagnosis of heart disease.

## Data Availability

The data analyzed in this study is subject to the following licenses/restrictions: In this study, two AF databases from different sources, i.e., ECG recordings from PhysioNet/CinC challenge 2017 and ECG PDFs from OUHSC, are used to evaluate the performance of data-driven detection methods. PhysioNet/CinC challenge 2017 is open-source database. The ECG PDFs from OUHSC are provided by our cardiologist collaborators. Requests to access these datasets should be directed to Stavros-Stavrakis@ouhsc.edu.
